# Determinants of Excessive Screen Time among Children under Five Years Old in Selangor, Malaysia: A Cross-Sectional Study

**DOI:** 10.3390/ijerph19063560

**Published:** 2022-03-17

**Authors:** Diana Raj, Norafiah Mohd Zulkefli, Zalilah Mohd Shariff, Norliza Ahmad

**Affiliations:** 1 Department of Community Health, Faculty of Medicine and Health Sciences, University Putra Malaysia, Serdang 43400, Malaysia; diana.2382002@gmail.com (D.R.); norafiah@upm.edu.my (N.M.Z.); 2 Ministry of Health Malaysia, Putrajaya 62590, Malaysia; 3 Department of Nutrition and Dietetics, Faculty of Medicine and Health Sciences, University Putra Malaysia, Serdang 43400, Malaysia; zalilahms@upm.edu.my

**Keywords:** screen time, child, television, parents, Malaysia

## Abstract

Excessive screen time interferes with the health and development of children. However, the screen time situation among Malaysian children remains poorly understood. This study aims to identify the prevalence and determinants of excessive screen time among children under five years in Selangor, Malaysia, using the latest World Health Organization guidelines. In this cross-sectional study, 489 parent–child dyads were randomly selected from nine government health clinics in Petaling district, Selangor. Total screen time and factors were assessed using validated self-administered questionnaires and analysed using multiple logistic regression. The overall prevalence of excessive screen time was 91.4% with a median of 3.00 h. The majority of children utilized television (66%), followed by handheld devices (30%) and computers (4%). Determinants of screen time identified were Malay ethnicity, (aOR 3.56, 95% CI 1.65–7.68), parental age of ≥30 years (aOR 3.12, 95% CI 1.58–6.16), parental screen time >2 h a day (aOR 2.42, 95% CI 1.24–4.73), moderate self-efficacy to influence a child’s physical activity (aOR 2.29, 95% CI 1.01–5.20) and the positive perception on the influence of screen time on a child’s cognitive wellbeing (aOR 1.15, 95% CI 1.01–1.32). Parents play an important role in determining their child’s screen time. Future interventions should focus on addressing parental determinants to ensure age-appropriate screen time.

## 1. Introduction

Screen time refers to the time spent on screen-based activities including television, smart phones, computers, tablets, video games and other handheld or visual devices [1]. Currently, the Malaysian Dietary Guidelines for Children and Adolescents (2013) recommends less than two hours of screen time for children in general, with no specific guidelines on screen time for children below five years of age [2]. However, in 2019, the World Health Organization (WHO) recommended no screen time for children below the age of two, whilst those aged between two to below five should be limited to one hour per day [3].

Globally, around 70% to 90% of children aged below the age of five did not meet the screen time guidelines [4,5,6,7,8,9]. Although excessive screen time is more prevalent among children from developed countries than developing countries, the subject will need to be interpreted with caution due to the scarcity of studies in the latter. Nonetheless, findings from a population-based study in India revealed that the prevalence of screen time among children below five was indeed high at 73% [10]. In Malaysia, the National Health and Morbidity Survey (NHMS) conducted in 2016 using the older American Academy of Pediatrics (1999) guidelines revealed that 52.2% of children below five years old exceeded the two-hour screen-time limit, with 74% of them aged below two years and 32.6% aged between two to below five years [11]. However, the determinants of excessive screen time among these children were not explored, which could be essential in creating targeted intervention programs. This figure is also expected to increase with the use of the latest WHO guidelines due to stricter recommendations.

Children who exceed the screen time recommendation are classified as having excessive screen time. This phenomenon has been largely exacerbated with the recent Coronavirus pandemic and multiple lockdowns imposed by the country. Excessive screen time has been associated with higher risks of developmental delay, particularly language delay, reduced physical activity, childhood obesity, hyperactivity-inattention, irritability, low mood and disrupted cognitive and socioemotional development, leading to poor educational performance, as well as limiting children using their imagination or exploring the world around them [10,12,13,14].

Although studies have been conducted to understand the factors associated with excessive screen time, it has mainly focused on school-age children [15]. It is insufficient to generalize evidence derived from older children to children aged below five years old due to developmental differences, as well as different influences towards their sedentary behaviours. Moreover, being a multicultural nation, a combination of cultural forces, and environmental factors provide a complex matrix of parental beliefs and parenting styles among Malaysian parents, which could also influence their child’s screen time. To the best of our knowledge, there have been no studies exploring the factors associated with screen time among children below five years in Malaysia. The aim of this study was to measure the prevalence of excessive screen time among children below five years old in Selangor, Malaysia, using the updated WHO 2019 guidelines, and to identify its determinants.

## 2. Materials and Methods

### 2.1. Study Sample

A cross-sectional study was conducted in the district of Petaling, Selangor, Malaysia. Selangor is the most populated state in Malaysia, with the Petaling district home to the highest number of children below five years old [16]. Data was collected from parent–child dyads of children below five years old, who were recruited from nine child health clinics between April and May 2019 using stratified sampling methods with a probability proportionate to clinic size. Based on the appointment book, a list of children below and above two years was made to ensure the sample was as representative of the population as possible. This was in assumption that the attendance of children aged below two will exceed those between two-five, due to the compulsory immunization schedule which stops at 18 months. The number of children sampled from each category followed the Malaysian population ratio of children aged below two and those aged between two to below five, which was 40% and 60%, respectively. Subsequently, each respondent was selected using a systematic sampling method, whereby the k interval of 14 was obtained by dividing the population size with the sample size. The first patient was selected using a random number generator, giving all respondents an equal chance of being selected. Children with any physical, mental, or chronic diseases were excluded from the study. The sample size was calculated using the two proportions method by Lawanga [17], based on previous studies comparing proportions of excessive screen time per day among children with siblings (63%) and without siblings (47%) [18]. A minimum sample size of 501 parents–child dyads was obtained upon adjusting for non-response rate of 30%, with alpha levels set at 0.05 and beta levels set at 0.20, respectively. 

### 2.2. Study Variables

A validated and reliable questionnaire was used during data collection [19,20,21,22,23,24,25], whilst height and weight measures were obtained from the child’s health record books. This questionnaire was pretested among 50 parents of children below five years old at one of the study sites; these samples were not included in the main study.

### 2.3. Dependent Variable

Screen time: Parental report of the total screen time was adapted from a study by Bernard et al. [26]. The child’s screen time per weekday and weekend were reported for three types of devices; (1) television (including but not limited to videos, DVD’s, PlayStation, Wii, Xbox), (2) computers (desktop and laptop) and (3) other handheld devices (including but not limited to mobile phones, tablets, iPad’s, Gameboy), before being averaged to obtain device-specific screen time in hours per day ([weekday × 5 + weekend day × 2]/7). Total screen time was calculated as the sum of time for all three types of devices. Children below two years old who had any screen time and children between two to below five years of age who had more than one hour of screen time were considered as having excessive screen time [3].

### 2.4. Independent Variable

Sociodemographic variables, including number of siblings and type of childcare setting (homecare by parents, non-parental homecare and center-based care), were adapted based on previous studies [18,27]. Body mass index (BMI) z-score was calculated based on the height (m) and weight (kg) obtained from the child health record book. The physical household environment was measured using three items, including the number of screen devices present in the household, whether screen devices (televisions, computers or handheld device) were present in the child’s bedroom (yes/no) and the presence or absence of outdoor play equipment [18,19,28]. Neighbourhood environment was assessed using three items, including availability of public facility such as park or playground (yes/no) for physical activity [29]. Perceived safety related to crime and perceived safety related to pedestrian walking was assessed using an adapted questionnaire from the Neighbourhood Environment Walkability Scale (NEWS) [20]. Responses ranged from (1) “strongly disagree” to (5) “strongly agree”. Scores were averaged to obtain thresholds of safety. Each unit increase in score revealed a lower threshold for both crime and pedestrian walking safety. Internal consistency for perceived safety related to crime was 0.9, whilst safety related to pedestrian walking was 0.8. 

Parental attitude towards screen time was made up of 8 items adapted from Carson and Jensen [19] and Asplund et al. [21], with responses ranging from (1) “strongly agree” to (5) “strongly disagree”. Average score of above 3 was categorized as having positive attitude while those scoring below 3 were classified as negative attitude. Internal consistency of items in this study was 0.84. Parents were also asked to indicate their self-efficacy in influencing their child’s physical activity level ranging from (1) “not confident” to (5) “very confident”. Scores were summed up to create a ‘self-efficacy to reduce child’s physical activity score scale’ and averaged to obtain the mean and median. A categorical variable was then derived by splitting the scores into three groups which were “low”, (25th percentile or less) “moderate” (between more than 25 to less than 75th percentile) and “high self-efficacy” (75th percentile or more). These items had an internal consistency of 0.88 [22]. Parenting style was assessed using an adapted version of the Steinberg instrument [23]. Two parenting styles dimensions were measured, namely “involvement” and “strictness” of parents. The involvement scale consisted of nine items with an internal consistency of 0.75, whilst the strictness scale was made up of six items, with an internal consistency of 0.78. Parents responded to the questions on a 5-point scale ranging from (1) “Strongly disagree” to (5) strongly agree” for all 15 items. For interpretation purposes, parents were categorized into the four parenting styles which is authoritative, authoritarian, indulgent and neglectful based on median splits of the “involvement” and “strictness” scale. 

Parents’ barriers to reduce screen time were obtained using a 6-item questionnaire adapted from Carson and Jensen [19] with an internal consistency of 0.78. Items were rated on a 5-point Likert scale ranging from (1) “Strongly disagree” to (5) strongly agree”. All responses were averaged to create an overall barrier score, whereby each unit increase in score reflected more barriers. Parents’ screen related restrictive practices were assessed on a Likert scale of “1–5” adapted from Pearson et.al with internal consistencies ranging between 0.81 and 0.83 [24]. Scores were summed with each unit increase in scores indicating more restrictive practices. Parents’ perception regarding the influence of screen time on their child’s well-being was assessed based on 11 health aspects that were classified into physical, cognitive and social wellbeing adapted from Hinkley et al. [25]. Questions were rated on a “3-point scale” with “positive influence” given a score of “3”, “no influence” given a score of “2” and “negative influence” given a score of “1”. One unit increase in parental perception score indicated greater perception of positive influence of screen time on child’s wellbeing. The internal consistency was 0.81.

### 2.5. Statistical Analysis

Analysis was conducted using the International Business Machines Statistical Package for the Social Science version 25.0. Descriptive statistics were used to describe the sample using frequency (*n*) and percentage (%) for categorical variable and mean and standard deviation (SD) or median and interquartile range (IQR) for continuous variables. Pearson’s chi-square test and simple logistic regression was used to determine the association between excessive screen time and its associated factors. Potential predictors of excessive screen time were screened to identify factors associated with excessive screen time in unadjusted models. A total of 11 independent variables with a *p* value of less than 0.25 [30], including those that were statistically significant, were chosen from the bivariate analysis to be included in the multivariate analysis. Subsequently, multiple logistic regression was conducted using the ‘Backward’ selection approach. Logistic regression included 95% confidence interval (95% CI). Significance level was set at alpha less than 5%.

## 3. Results

Out of the 510 questionnaires distributed to eligible parents, 489 consented and completed the questionnaire, yielding a response rate of 96%. The overall prevalence of excessive screen time among children below five years old in this study was 91.4%. Children in this study spent an average of 3 h (IQR 1.36–5.04) per day watching screens, where the majority spent time watching television (66%), followed by handheld devices (30%) and computers (4%). The distribution of screen time among children aged below 2 years and those aged be-tween 24–59 months is illustrated in Figure S1.

The mean age of parents in this study was 32.2 ± 0.2 years. More than half of them earned a monthly household income of less than Ringgit Malaysia (RM) 5000 (56.6%) and were employed (78.3%). Gender of the children who participated in the study were of almost equal percentage, with 51.7% of them being male and were taken care for by their parents only (56.4%). Table 1 shows the association between sociodemographic, household and neighbourhood characteristics and screen time of the children. Older parents aged 30 years and above, children from the Malay ethnicity, presence of siblings, and having outdoor play equipment for the child to play with were associated with excessive children’s screen time.

Table 2 shows the association between parental factors and screen time, whereby parents own screen time was significantly associated with their child’s excessive screen time (*p* = 0.002).

The results of the multivariate analysis (Table 3) showed that children from the Malay ethnicity were more likely to have excessive screen time (aOR = 3.56, 95% CI 1.65–7.68) compared to other ethnicities. Children who had parents aged 30 years and above were more likely to have excessive screen time (aOR = 3.12, 95% CI 1.58–6.16) as compared to those who had parents aged less than 30 years. Parental screen time of more than 2 h a day was the strongest modifiable predictor of excessive screen time among children aged below five years (aOR = 2.42, 95% CI 1.24–4.73). Children whose parents had moderate self-efficacy to influence a child’s physical activity were 2.3 times more likely to have excessive screen time compared to parents who had higher self-efficacy (aOR = 2.29, 95% CI 1.01–5.20). For one-unit increase in parental perception score of positive influence of screen time on their child’s cognitive wellbeing, there was a 1.2 unit increase in screen time (95% CI 1.01–1.32).

## 4. Discussion

This study was conducted to assess the prevalence of excessive screen time among children below five years of age and its determinants. Overall results indicate that more than 90% of children below five years of age in Selangor, Malaysia exceeded the WHO age-appropriate screen time limit. The prevalence is higher than studies conducted in developed countries utilising similar guidelines such as Canada (62%) [31]. However, meaningful comparisons between other countries are difficult due to the lack of uniformity in guidelines prior to this. In our analysis, we found a positive association between excessive child’s screen time and parental factors—ethnicity, parental age, parental screen time, parental self-efficacy to influence child’s physical activity and parent’s perception on the positive influence of screen time on child’s wellbeing. These findings are consistent with previous studies [28,32,33,34].

An interesting finding of this study is the role of ethnicity in a child’s screen time. Children from the Malay ethnicity were more than three times likely to have excessive screen time compared to other ethnicities. The fertility rate in 2018 for Malay women of a childbearing age in Malaysia was 2.4 births per one thousand women, compared to those of Indian and Chinese ethnicity with 1.3 and 1.1, respectively [35]. With the increased participation of women in the labour workforce [36], having a large family in addition to a job, family, and dual working parents, multitasking can be challenging. Furthermore, adults within 30–45 years have been labelled as the sandwich generation, having to also include elderly care into their duties. Adhering to the responsibility of providing care for both children and the elderly, as emphasized by Malay culture, can increase stress among caregivers [37]. This could explain why screens become a relief in childrearing among the Malays.

In addition, screen time is also known to be inversely associated with levels of physical activity [38]. Studies have shown that those of a Malay ethnic background were less likely to engage in physical activities compared to other ethnicities [39,40]. In addition, the parenting style and worry about their child’s safety has been shown to be a prominent factor that influences Malay parents’ decisions. A study among Malaysian pre-schoolers showed that a significantly higher proportion of Malay parents (49.9; 95% CI 44.2–55.7) expressed worry about their child’s safety against crime and injury when participating in physically active play, compared with Chinese parents (30.6; 95% CI 23.6–38.6) [41]. This in turn could be a potential contributor to the increase in Malay children resorting to sedentary screen time activities indoors.

Children of older parents aged above 30 years were three times more likely to have excessive screen time compared to those aged below 30 years. This was similar to findings from a cohort study in Finland [42]. Maternal age has shown to have a significant influence on the parenting attitude and behaviours of mothers. Older mothers with previous experience as a parent could also become more focused on other self-actualization activities beyond the home [43]. These could be reasons why older parents do not prioritize good screen time parenting behaviours, leading to an increase in screen time among these children. Furthermore, the majority of older parents in this study had more than one child and there could be a possibility of needing a coping tool to meet the demands of raising multiple children [44]. 

Parents played an important role in modelling behaviours to their children [34]. Studies have shown that parents who limit their own screen time have seen a significant reduction in their child’s screen time as well. The Malaysian Communications and Multimedia Commission reported that the percentage of Malaysian adults using the internet via screens was 88.7%, with the majority spending 5–12 h per day [45]. Intriguingly, the majority of them used screen time for social purposes instead of work-related activities, with an increase in online gaming among 42.8% of users. Moreover, a large fraction of them reported taking part in these activities within the home environment. Predictably, children below five spend most of their time at home, observing the screen time behaviours of their parents. This may explain parental screen time being one of the determinants of excessive screen time among children below five years in this study. 

Our finding on the association between parents’ self-efficacy to influence a child’s physical activity and children’s excessive screen time was consistent with an Australian study, whereby relatively lower self-efficacy was associated with the likelihood of an increase in screen time [46]. A local study showed that Malaysian parents tend to display higher self-efficacy when it comes to nurturing and responsiveness compared to only moderate self-efficacy towards the establishment of routines and limited settings [47]. This may explain why establishing routines such as screen time schedules might be a more difficult task for parents. Self-efficacy was also related to their psychological and emotional states, whereby anxiety and stress would result in lower self-efficacy [48]. Likewise, more than half of the participating parents reported having no confidence to influence their child’s physical activity when they were feeling stressed. 

When parents perceive that screen time can contribute to cognitive benefits, they tend to encourage and not limit the time spent on screens, therefore giving their children more access to screen devices [13]. However, parental perception on cognitive wellbeing in this study did not play a significant role compared to previous studies [25,49], suggesting that there may be other pressing reasons why parents allowed excessive screen time as discussed above. 

This study is among the first to use classifications from the recently published WHO 2019 children screen time guidelines [3]. It contributes to the lack of locally existing research on excessive screen time among children aged below five years old in Malaysia, using a wide range of variables. Data obtained in this study serves as a baseline for future comparable or interventional research in this area. However, potential limitations need to be noted. The cross-sectional nature of the study limits drawing inferences on cause and effect. The use of self-administered questionnaires might have also led to inconsistency with actual experience. Moreover, parental reports of their child’s screen time might be subjected to recall bias of their child’s actual screen time. Parents may also be influenced by social desirability, both in terms of their child’s screen time behaviour as well as to present themselves in ways that they believe are expected of them as parents. This could lead to under or over-reporting. Furthermore, given that this study was performed among parents who completed their children’s health schedules in a particular district, findings from this study need to be interpreted with caution in view of the limited generalizability to other populations.

## 5. Conclusions

The main determinants for excessive screen time among children under-five years old were ethnicity, parental age and other parental factors such as parent’s screen time, parent’s self-efficacy to influence a child’s physical activity and parent’s perception of the influence of screen time on a child’s well-being. Interventions that aim to address these factors may foster healthy screen time habits in children.

## Figures and Tables

**Table 1:** Association between sociodemographic, household and neighbourhood characteristics and children screen time (*n* = 489).

	Descriptive Statistics			Bivariate Analysis		
Variable	*n* (%)	Excessive Screen Time (%)	LowScreen Time (%)	*χ*^2^ (*df*)	Β (95% CI)	*p* Value
**Parents Age**				10.65 (1)		0.001 ^c^
<30 years	148 (30.3)	126 (85.1)	22 (14.9)			
≥30 years	341 (69.7)	321 (94.1)	20 (5.9)			
**Parents Education Level**				0.001 (1)		0.98
Lower Education	164 (33.5)	150 (91.5)	14 (8.5)			
Higher Education	325 (66.5)	297 (91.4)	28 (8.6)			
**Monthly Household Income**				1.09 (1)		0.30
less than RM 5000	277 (56.6)	250 (90.3)	27 (9.7)			
RM 5000 and more	212 (43.4)	197 (92.9)	15 (7.1)			
**Employment status**				0.002 (1)		0.97
Employed	383 (78.3)	350 (91.4)	33 (8.6)			
Unemployed	106 (21.7)	97 (91.3)	9 (8.7)			
**Child’s Age**				0.03 (1)		0.96
<24 months	258 (52.8)	236 (91.5)	22 (8.5)			
24–59 months	231 (47.2)	211 (91.3)	20 (8.7)			
**Child’s Sex**				1.9 (1)		0.17
Male	253 (51.7)	227 (89.7)	26 (10.3)			
Female	236 (48.3)	220 (93.2)	16 (6.8)			
**Ethnicity**				10.37 (1)		0.001 ^c^
Malay	419 (85.7)	390 (93.1)	29 (6.9)			
Non-Malay	70 (14.3)	57 (81.4)	13 (18.6)			
**Marital Status** ^a^						0.42
Married	483 (98.8)	442 (91.5)	41 (8.5)			
Divorced/Widowed/Separated	6 (1.2)	5 (83.3)	1 (16.7)			
**Presence of Siblings**				6.05 (1)		0.01 ^c^
No	182 (37.2)	159 (87.4)	23 (12.6)			
Yes	307 (62.8)	288 (93.8)	19 (6.2)			
**Childcare settings**				1.26 (2)		0.53
Parental care only	276 (56.4)	249 (90.2)	27 (9.8)			
Home based childcare	104 (21.3)	96 (92.3)	8 (7.7)			
Childcare centers	109 (22.3)	102 (93.6)	7 (6.4)			
**BMI z-Score**				0.33 (1)		0.57
Not overweight	416 (85.1)	379 (91.1)	37 (8.9)			
Overweight/Obese	73 (14.9)	68 (93.2)	5 (6.8)			
**Total Gadgetsat home** ^a^						0.99
<3 gadgets	35 (7.2)	32 (91.4)	3 (8.6)	-		
3 or more gadgets	454 (92.8)	415 (91.4)	39 (8.6)			
**TV in Bedroom**				0.55 (1)		0.46
Yes	74 (15.1)	66 (89.2)	8 (10.8)			
No	415 (84.9)	381 (91.8)	34 (8.2)			
**Outdoor Play Equipment** ^a^						0.03 ^c^
No	29 (5.9)	23 (79.3)	6 (20.7)			
Yes	460 (94.1)	424 (92.2)	36 (7.8)			
**Public facility (i.e., park)** ^a^						0.57
No	44 (9.0)	39 (88.6)	5 (11.4)			
Yes	445 (91.0)	408 (91.7)	37 (8.3)			
**Crime Safety** ^b^	3.71 ± 0.04				0.81 (0.57–0.1)	0.23
**Pedestrian safety** ^b^	3.13 ± 0.03				0.72 (0.45–1.18)	0.19

Values represent mean (SD) for continuous values; frequency and percentage for categorical values; ^a^ Fishers exact test; ^b^ simple linear regression; ^c^ Significant at *p* < 0.05.

**Table 2:** Association between parental factors and children screen time.

Variable	Descriptive Statistics			Bivariate Analysis		
	*n* (%)	Excessive Screen Time (%)	Low Screen Time (%)	*χ*^2^ (*df*)	Β (95% CI)	*p* Value
**Attitude towards screen time**				0.33 (1)		0.57
Negative	306 (37.4)	278 (90.8)	28 (9.2)			
Positive	183 (62.6)	169 (92.3)	14 (7.7)			
**Parenting Style**				0 (1)		>0.99
Authoritative	163 (33.3)	149 (91.4)	14 (8.6)			
Non authoritative	326 (66.7)	298 (91.4)	28 (8.6)			
**Self-Efficacy**				4.89 (2)		0.09
Low SE	101 (20.7)	88 (87.1)	13 (12.9)			
Moderate SE	274 (56.0)	257 (93.8)	17 (6.2)			
High SE	114 (23.3)	102 (89.5)	12 (10.5)			
**Parental Screen Time**				9.52 (1)		0.002 ^b^
2 h or less	173 (35.4)	149 (86.1)	24 (13.9)			
More than 2 h	316 (64.6)	298 (94.3)	18 (5.7)			
**Perception on wellbeing** ^a^	23.03 ± 0.24				1.03 (0.97–1.09)	0.27
Physical	8.00 ± 4.0				0.99 (0.86–1.14)	0.88
Cognitive	10.0 ± 4.0				1.11 (0.98–1.26)	0.09
Social	6.0 ± 3.0				1.08 (0.92–1.27)	0.34
**Barriers ^a^**	2.95 ± 0.03				0.83 (0.52–1.34)	0.45
**Restrictive practices** ^a^	26.6 ± 0.17				1.04 (0.95–1.14)	0.39

Values represent mean (SD)/median [IQR] for continuous values, and frequency and percentage for categorical values; ^a^ Simple linear regression; ^b^ Significant at *p* < 0.05.

**Table 3:** Determinants of excessive screen time among children below five years of age.

Variable	^a^ OR (95% CI)	^b^ aOR (95% CI)	*p* Value
**Parent’s Age**			
<30 years	1	1	
≥30 years	2.80 (1.48–5.31)	3.12 (1.58–6.16)	0.001 ^c^
**Ethnicity**			
Non-Malay	1	1	
Malay	3.07 (1.51–6.24)	3.56 (1.65–7.68)	0.001 ^c^
**Parental Perception on influence of screen time on child’s cognitive well-being**	1.13 (0.98–1.26)	1.15 (1.01–1.32)	0.04 ^c^
**Parental Self-Efficacy to influence child’s physical activity**			
High	1	1	
Low	1.26 (0.55–2.89)	0.96 (0.40–2.31)	0.92
Moderate	2.23 (1.04–4.78)	2.29 (1.01–5.20)	0.047 ^c^
**Parent’s screen time**			
2 h or less	1	1	
more than 2 h	2.67 (1.40–5.07)	2.42 (1.24–4.73)	0.01 ^c^

^a^ Simple logistics regression; ^b^ Multiple logistic regression; ^c^ Significant at *p* < 0.05; aOR = adjusted Odds Ratio.

## Data Availability

Data supporting findings of this study are available upon reasonable request to the corresponding author N.A.

## Supplementary Materials

The following are available online at https://www.mdpi.com/article/10.3390/ijerph19063560/s1, Figure S1: Distribution of screen time among children below 5 years.
