# Supplementary figures and images for: Determinants of Excessive Screen Time among Children under Five Years Old in Selangor, Malaysia: A Cross-Sectional Study
Source: Int J Environ Res Public Health. 2022 Mar 17;19(6):3560. doi: 10.3390/ijerph19063560 (PMC8951484; doi:10.3390/ijerph19063560)

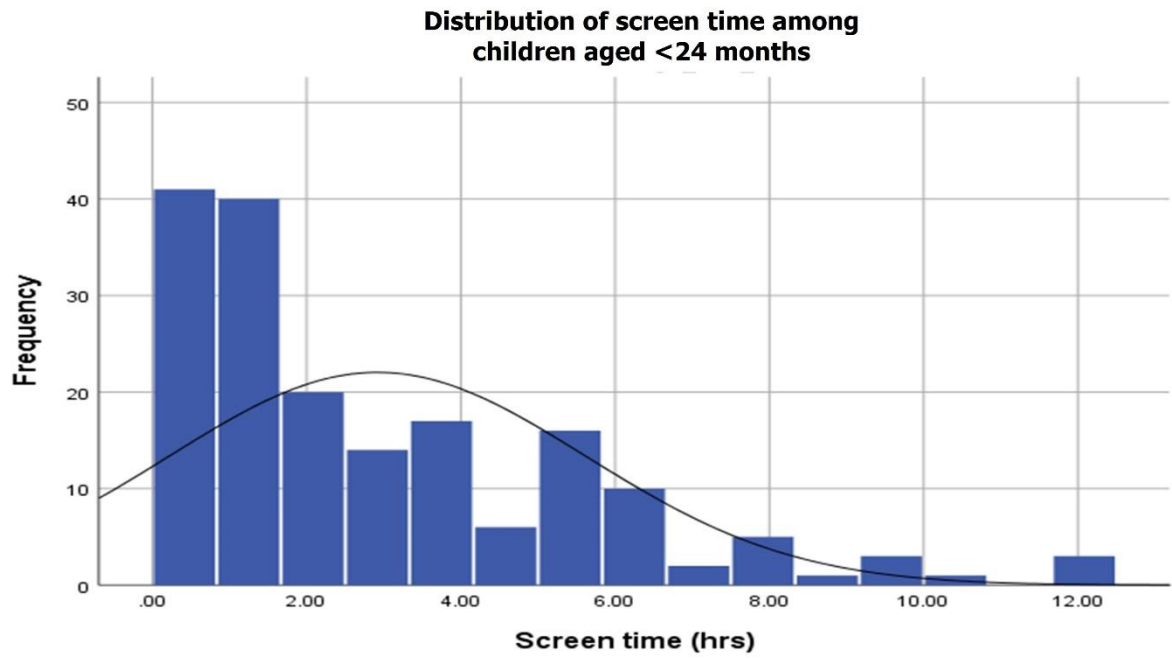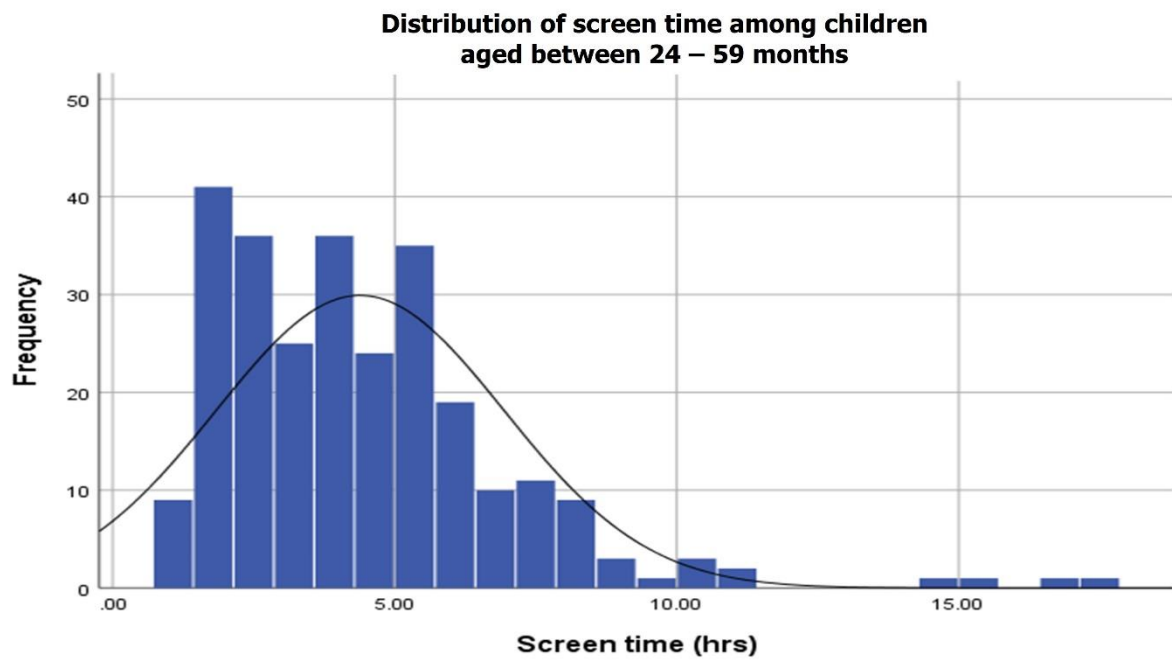

Figure S1. Distribution of screen time among children below 5 years.

Supplement: Supplementary file 1 [file ijerph-19-03560-s001.zip › ijerph-1587199-supplementary.pdf]
